# Supplementary material for: Synergy between Indoloquinolines and Ciprofloxacin: An Antibiofilm Strategy against Pseudomonas aeruginosa
Source: Antibiotics (Basel). 2021 Oct 4;10(10):1205. doi: 10.3390/antibiotics10101205 (PMC8532862; doi:10.3390/antibiotics10101205)
Supplement: Supplementary file 1 [file antibiotics-10-01205-s001.zip › antibiotics-1381430-supplementary.pdf]

Supplementary Material

Synergy between Indoloquinolines and Ciprofloxacin: An Antibiofilm Strategy against *Pseudomonas aeruginosa*

Emilie Charpentier [1], Ludovic Doudet [2], Ingrid Allart-Simon [2], Marius Colin [1], Sophie C. Gangloff [1], Stéphane Gérard [2,†] and Fany Reffuveille [1,†,*]

[1] Université de Reims Champagne-Ardenne, EA 4691 Biomatériaux et Inflammation en Site Osseux (BIOS), SFR Cap Santé (FED 4231), UFR Pharmacie, 51 rue Cognacq-Jay, F-51096 Reims, France.; emilie.charpentier@univ-reims.fr (E.C.); marius.colin@univ-reims.fr (M.C.); sophie.gangloff@univ-reims.fr (S.C.G.)

[2] Université de Reims Champagne-Ardenne, Institut de Chimie Moléculaire de Reims (ICMR), UMR CNRS 7312, UFR Pharmacie, 51 rue Cognacq-Jay, F-51096 Reims, France. ludovic.doudet@univ-reims.fr (L.D.); ingrid.allart-simon@univ-reims.fr (I.A.-S.); stephane.gerard@univ-reims.fr (S.G.)

* Correspondence: fany.reffuveille@univ-reims.fr

† These authors contributed equally to this work.

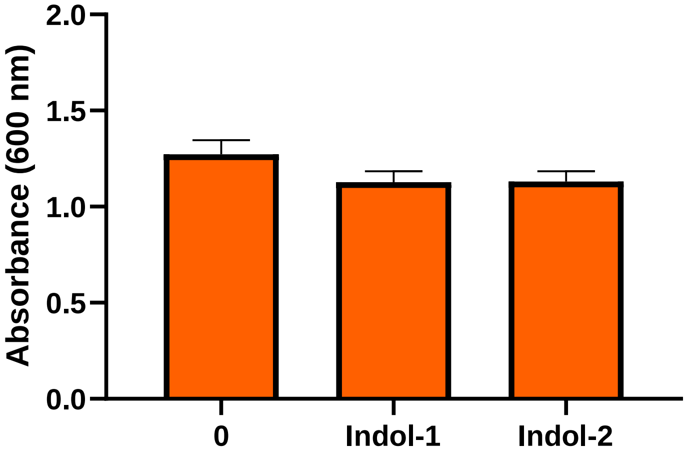

Figure S1. Indol-1 and Indol-2 treatments of *P. aeruginosa*. The graph represents planktonic growth after 24 h incubation in presence of 40 µg/mL of Indol-1 and Indol-2 molecules (absorbance at 600 nm).
